# Virtual reality for pain management: an umbrella review

**DOI:** 10.3389/fmed.2023.1203670

**Published:** 2023-07-14

**Authors:** Dmitriy Viderman, Karina Tapinova, Mukhit Dossov, Serik Seitenov, Yerkin G. Abdildin

**Affiliations:** ^1^School of Medicine, Nazarbayev University, Astana, Kazakhstan; ^2^Department of Anesthesiology and Intensive Care, National Research Oncology Center, Astana, Kazakhstan; ^3^Department of Anesthesiology and Critical Care, Presidential Hospital, Astana, Kazakhstan; ^4^School of Engineering and Digital Sciences, Nazarbayev University, Astana, Kazakhstan

**Keywords:** virtual reality, pain control, chronic pain, postoperative pain, acute pain

## Abstract

**Background and objective:**

Virtual reality is a promising pain control strategy for various pain conditions. This umbrella review of systematic reviews and meta-analyses aims to evaluate the analgesic effects of virtual reality.

**Methods:**

We searched for the relevant reviews in Scopus, PubMed and Cochrane library. Our primary outcome was pain, with secondary outcomes including disability, general health status, patient satisfaction, depression, balance, fear of movement, and adverse events. The quality of included articles was evaluated using the AMSTAR-2 tool.

**Results:**

21 systematic reviews and meta-analyses with 274 studies and 17,680 patients were included in this review. All the reviews concluded benefits of virtual reality in managing pain conditions, including chronic and pain.

**Discussion and conclusions:**

This umbrella review demonstrates successful application of virtual reality in pain control, including perioperative, periprocedural, and chronic pain settings. Virtual reality can be used as an alternative therapy for pain management in children and adults.

## 1. Introduction

Virtual reality (VR) has tremendously advanced in the last 20 years (1, 2). It has gained increasing interest in recent years for its potential in managing pain. VR has been defined as “simulations that make use of various combinations of interaction between devices and sensory display systems” (3). Recently, the use of VR has extended to various clinical fields, such as physical rehabilitation, pain management, and different neurological and psychiatric disorders. It is a non-invasive, drug-free, and user-friendly analgesic approach (1–3). VR has been explored in neurological conditions including Parkinson’s disease, phobias, and pain in phantom limbs following amputations (3). VR has also served patients with pain and physical disability, with higher anxiety, and those seeking an alternative to opioid analgesics (4–6). Generally, recent systematic reviews (SR) examining the effectiveness of this technology pointed out its potential to manage a variety of pain-associated conditions (5). A recently published SR reported that VR was beneficial in burn patients alleviating anxiety and pain when undergoing physical rehabilitation (7). Similarly, another SR found that VR could bring benefits to patients with burns, eating disorders, and traumatic brain injury (8). VR offers an immersive experience that can simulate various environments. The suggested mechanisms of VR in pain management mainly include heightened distraction with gaining patient’s attention and activating many senses (3). Virtual environments use head tracking systems, tactile feedback and highly stimulating visual and auditory sensations. In this way, the individual becomes immersed in the virtual world, taking attention away from the perception of pain (3). It was hypothesized that the VR program, as a distractor, reduces the conscious sensation of pain through the signaling pathways leading to pain (9). Therefore, virtual reality may alter the behavior of the pain modulation system by lowering the concentration on pain resulting in a stimulus not being perceived as painful. By activating the visual cortex and integrating additional senses, virtual reality alters the handling of nociceptive stimuli. Brain functional magnetic resonance imaging (fMRI) confirms that virtual reality influences the insular and sensory cortex similar to opiates. Being expensive, VR systems have limited use, but with the increasing use of high-resolution mobile phones, VR has become a more adaptable and accessible tool for pain relief (10, 11).

This non-invasive and drug-free approach to pain management makes it a promising alternative to traditional methods. Since the number of clinical trials, as well as systematic review and meta-analyses (SR&MA) exploring the possibilities of VR in pain medicine is rapidly growing, to examine the highest level of evidence, we conducted an umbrella review of SRs and MAs.

## 2. Methods

We performed a systematic search in Scopus, PubMed as well as “Cochrane database of systematic reviews” from inception to February 15, 2023, for SRs & MAs focusing on the effect of virtual reality in pain management. We then manually searched further the citations of the selected eligible papers to find other publications, which we might have overlooked during the original search. To prepare this SR we followed “the Preferred Reporting Items for Systematic Reviews and Meta-analyses (PRISMA) recommendations” (12).

### 2.1. Search strategy

We conducted a systematic search in Scopus, PubMed, and Cochrane library. To find the eligible studies, we used the subsequent search terms: “virtual reality,” “virtual,” “reality,” “pain,” “anxiety,” “anxieties,” “anxiety s.”

We designed the following inclusion criteria to identify the available evidence in the research area:

1. Systematic reviews and meta-analysis;
2. Studies that involved VR for pain management;
3. Studies published in full in peer-reviewed journals.

Exclusion criteria:

Not SR&MA (observational studies, randomized controlled trials, animal studies, editorials; correspondence).

### 2.2. Outcomes

The primary outcome was an assessment of pain intensity. Disability, general health status, patient satisfaction, depression, and anxiety were included as secondary outcomes.

### 2.3. Data extraction, data analysis, and quality assessment

We extracted the following data in the tables for the subsequent analysis: (1) author, citation; (2) study design; (3) study goals; (4) the number of patients included in each MA; (5) the total number of studies included in MA; (6) diagnosis; (7) surgery, procedure, or pain condition; (8) study conclusions.

We evaluated the quality of the included SRs & MAs using the AMSTAR-2 (A MeaSurement Tool to Assess systematic Reviews) (13), using 16-criterion items that assess quality of the methodology of the systematic reviews, including study selection and screening, data extraction and synthesis, as well risk of bias assessment. The domains were rated as “yes,” “partially yes,” and “no.” The assessment of the overall confidence in a systematic review is based on domains 2, 4, 7, 9, 11, 13, and 15. High confidence in a review means that there are no weaknesses in these critical domains. Moderate confidence is assigned when the review has non-critical weaknesses. Low confidence means that there is at least one critical weakness. Critically low confidence is assigned when the review has more than one critical weakness.

## 3. Results

Twenty-one SR and MAs reporting the results of 274 original studies (excluding overlapping studies), 222 RCTs, and 52 observational studies were included (Table 1). The total number of patients excluding overlapping studies was 17,680.

**Table 1:** Study characteristics.

Author, citation	Study design	Study goals	N of patients included in MA	Total number of studies included in MA	Diagnosis	VR programs	Surgery, procedure, or pain condition	Study conclusions
Ahern (9)	SR + MA of RCTs	Primary – pain, secondary – function, doctor visits, return to work, patient satisfaction, AEs	311	7	Chronic spinal and neck Pain	Nintendo Wii sports program, VR-based computer game, airplane flight via headset, kinematic training via head-mounted laser, horse-riding simulation	Spinal and neck Pain	VR improves spinal pain
Baradwan (14)	SR + MA of RCTs	Primary – pain (VAS) Secondary – anxiety, satisfaction	466	8	Pregnant	NG	Labor	VR decreases anxiety and pain and improves satisfaction
Bordeleau (1)	SR + MA of RCTs and observational studies	Pain (VAS, NRS)	900	24	Chronic low back pain	VR-based exercises program, VR-based horse-riding simulator, VR-based graded exposure, CBT using SnowWorld, PlayMancer progressive exergame, ValedoMotion exercise system, Nintendo Wii exercise, Kinect games, ProKin 252 balance training, dodgeball games, VR-based motor imagery	Back pain	VR improves back pain and function
Chan (15)	SR + MA of RCTs and crossovers	Pain	656	16	Burns, wounds	HMD via PC, joystick; HMD via laptop, inertial tracking; HMD-mounted phone, gaze tracking; HMD via PC, trackball; HMD via DVD player; HMD via PC, mouse; HMD, keyboard/mouse. Interactive games and non-interactive videos	Port access, peripheral IV cannula, Venepuncture, Episiotomy repair, LP, cystoscopy, burns and wound care	VR can improve pain
Czech (16)	SR + MA of RCTs and crossovers	Pain, ROM, fear, anxiety	587	17	Burns, wounds	Ditto non-interactive video, immersive VR game, non-immersive VR relaxation, smartphone VR	Burns and wound care	VR improves pain
Eijlers (17)	SR + MA	Pain, anxiety	859	17	Burns, elective surgery, oncology	i-glasses, nVisor SX, ProView, eMagin Z800 3DVisor, VR-1280, Virtual IO, i-O Display Systems, HMD with tracker, Oculus Rift, Samsung Gear VR, Google Pixel, Merge VR	venous access, chemotherapy, LP, port access	VR improves pain and anxiety
Gao (18)	SR + MA of RCTs	Pain, anxiety, fear	2,224	27	-	NG	LP, IV, plastic surgery, port access, laceration repair	VR improves pain and anxiety
Gates (19)	SR + MA of RCTs, cross-sectional, and other observational studies	Pain	7,820	106	Burns, wounds	NG	IV, dental anesthesia, immunization	VR improves pain
Grassini (20)	SR + MA of RCTs	Pain (VAS), disability	524	9	Neck and low back pain	VR Dodgeball, Kinect Xbox 360: Fruit Ninja game, Cervi game, Video of walking down the Ireland forest, VR: kinematic training, VR multimedia with audio, VR Vox Play glass with HMD and smartphone, VR shooting game.	Neck and low back pain	VR improves chronic pain
He (21)	SR + MA of RCTs	Pain (VAS), BP	1,258	13	Burns, wounds, hemorrhoids, vascular ulcer, war blast wound, abdominal surgery	Ditto, Sony VR Play-station, 3D glasses, headphones, eMagin Z800 3DVISOR, headset.	Dressing change, hemorrhoids, abdominal surgery	VR improves pain
Huang (22)	SR + MA of RCTs	Pain (VAS)	1947	31	Breast cancer, hemorrhoids, fibromyalgia, injury, burns	Immersive VR, Snow World, Google Pixel XL/Google Daydream, iWear Video Headphones, Oculus DK2 HMD, ezVision X4 VR goggles, Virtual Gorilla program, Samsung Galaxy S6 mobile-based Gear VR goggles, Nintendo Wii eMagin Z800 3D VISOR HMD, Ditto	Hemorrhoidectomy, needle and dental procedures, lower limb amputation, low back pain, TKR, fibromyalgia	VR improves pain
Li (6)	SR + MA of RCTs	Pain, AEs	273	4	Wounds, burns	HMD, glasses	IV, dressing change	VR improves pain
Luo (23)	SR + MA of RCTs	Pain, anxiety, ROM, AEs	362	13	Burns	Immersive games	Dressing change, physical therapy	VR improves pain
Rajendram (24)	SR + MA	Pain (VAS)	300 (214/86) (mirror/VR)	15 (8/7) (mirror/VR)	phantom limb pain	NG	phantom limb pain	Mirror therapy and VR decrease pain
Simonetti (25)	SR + MA of RCTs	Anxiety, pain	602	7	-	HTC Vive visor, Oculus Rift Headset, Oculus Rift Headset, Samsung Gear VR headset	Before surgery	VR improves anxiety
Suleiman-Martos (26)	SR + MA of RCTs	Pain, anxiety	2,525	26	Hernia, rheumatology, ophthalmology, orthopedic	VR eyeglasses	Dental, ENT, circumcision, abdominal, urology, elective minor or major surgeries	VR improves anxiety
Tas (27)	SR + MA of RCTs, crossovers, and non-RCTs	Pain, anxiety	1,695	13	Burns, cancer	i-glasses; nVisor; ProView eMagin 3DVisor; Kaiser Optical; 5DT HMD; Oculus Rift DK2; Samsung Gear VR; Google Pixel; Merge VR	IV, port access, dressing change, physical therapy, dental, nasal endoscopy	VR improves pain and anxiety
Tran (28)	SR + MA of RCTs	Pain	438	10	Cancer	NG	IV, LP	VR improves pain
Zeng (29)	SR + MA of RCT, case–control, and other observational studies	Anxiety, pain	225	6	Cancer	Nintendo Wii Fit Plus, goggles	Cancer pain	VR improves pain and anxiety
Zhang (30)	SR + MA of RCTs and observational studies	Pain, anxiety, ROM	529	8	Breast cancer	Forearm support, HMD and headphones, movement tracking system, VR exploration on Second Life platform, Xbox360 Kinect video game, VERT system	Surgery, chemotherapy	VR improves pain

AE, adverse event; BP, blood pressure; HMD, head-mounted display; IV, intravenous; LP, lumbar puncture; MA, meta-analysis; NRS, numerical rating scale; RCT, randomized controlled trial; ROM, range of motion; SR, systematic review; TKR, total knee replacement; VAS, visual analogue scale; VR, virtual reality.

### 3.1. Patient characteristics

Chronic low back pain, neck pain, burn wounds, elective surgery, oncological patients (non-specified), hemorrhoids, vascular ulcer, war blast wound, abdominal surgery, breast cancer, hemorrhoids, fibromyalgia, phantom limb pain, hernia, rheumatology, ophthalmology, orthopedic, and obstetric surgery (Table 1).

### 3.2. Characteristics of surgery or procedure

Hemorrhoidectomy, needle and dental procedures, lower limb amputation, phantom limb pain, total knee replacement (TKR), intravenous injection, dressing change, physical therapy, dental procedures, ENT, circumcision, abdominal surgery, urological surgery, elective minor or major surgeries, port insertion, nasal endoscopy, and chemotherapy (Table 1).

### 3.3. Characteristics of VR

Among the VR programs used, visual and tactile sensations were encountered most frequently. Visual sensations were delivered through devices such as Head-Mounted Displays (HMDs), VR eyeglasses, and various models of VR goggles (like Oculus Rift, Samsung Gear VR, Google Pixel, Merge VR, etc.). These devices deliver immersive visuals for programs like games, airplane flight, and horse-riding simulation. Tactile sensations come into play through interfaces such as joysticks, trackballs, mouse, keyboard, motion sensors, and inertial tracking devices. As for the most used types of programs, game-based and exercise-based VR programs seem to dominate the list. Examples of game-based programs include the VR Dodgeball and Fruit Ninja game. Exercise-based programs are represented by the Kinect games, balance training, motor imagery, and other exercise programs.

### 3.4. VR efficacy in managing chronic pain

All 21 SRs & MAs reported positive results. Of them, 10 studies reported positive results in improving pain, four reported improvements of both pain and anxiety, two studies reported improvement in anxiety, one reported improvement in both pain and function, and one reported improvement in mental health.

### 3.5. VR in chronic low back and neck pain

Patients with chronic spinal and neck pain were treated using a variety of VR exercise programs. Although the use of VR therapy in clinical settings has been rapidly advancing, there is limited evidence to prove its benefit for chronic pain management. However, previous studies and meta-analysis suggest that interventions involving virtual reality may be beneficial in managing chronic pain, particularly in the case of neck pain, and reducing neck disability in comparison to control groups. Nonetheless, it is not clear yet if VR therapy is more effective than other types of pain management treatments.

VR has some room for improvement in spinal pain patients. It might result in statistically and clinically significant improvement in the following outcomes: pain scores, disability, patient satisfaction, kinesiophobia, balance, and overall health condition. Higher-quality and more focused studies are still needed (9).

### 3.6. VR in pain relief during labor

VR can effectively reduce anxiety and reduce painful sensations in women during labor (14). A significant percentage of women who tried VR claimed it to be more satisfied and expressed interest in using VR in future labor. VR might be a preferred method for pain management during normal labor due to its non-pharmacological nature, cost-effectiveness, and absence of side effects. VR can improve the quality of healthcare service and patient comfort. VR might assist women in relaxing while giving birth (10).

### 3.7. VR during painful procedures including wound care

Programs for wounds and burns included both interactive games and non-interactive videos (16, 23). VR can be used as an additional method to reduce pain levels during painful procedures. VR intervention can decrease a patient’s perception of pain, emotional response to pain, and blood pressure during wound care (21).

### 3.8. VR in phantom limb pain patients

VR might be effective in reducing phantom limb pain (PLP). VR therapy provided advantages over mirror therapy, including greater freedom to manipulate the missing limb as in the standard therapy, movements are limited to the intact and phantom limbs moving simultaneously. The VR system is capable of incorporating time lags to create the impression that the virtual limb moves independently of the present one. Unlike mirror therapy, VR therapy can be used in those with both limbs missing since it does not require a present limb to be reflected; instead, the movements of the patient’s stump can be tracked (24).

### 3.9. VR in pediatrics

The use of virtual reality distraction was shown to provide a significant effect on reducing pain-and anxiety-related stress in pediatric patients during clinical interventions, particularly in younger children. It was reported to be a user-friendly tool that could be effectively applied in hospital settings. However, further experiments are necessary to establish conclusive evidence on the effectiveness of VR in reducing pain and anxiety during medical procedures in pediatrics (17–19, 25–27).

VR can help make children less anxious and fearful and feel less discomfort when undergoing needle procedures. It can be recommended during puncture procedures as a useful way to alleviate negative emotions in pediatric patients (18, 19).

### 3.10. VR during the perioperative period in pediatrics

VR is an effective method for making pediatric patients less anxious before elective surgery (17). Virtual reality could become a useful nonpharmacological intervention to address preoperative anxiety in young patients and improve their hospital experience without requiring significant resources.

Additionally, virtual reality has the potential to positively impact recovery by reducing anxiety and improving pain perception during the postoperative period. This could result in better outcomes, such as reduced morbidity and hospitalization times, and lower related health costs.

The use of game-based VR to alleviate preoperative anxiety in children has a positive effect, as found by our analysis, but it does not significantly impact pain levels. This type of intervention is enjoyable and innovative, and it can help nursing professionals provide positive attention to pediatric patients undergoing surgery. By reducing anxiety and pain during preoperative care, game-based interventions can benefit a child’s emotional health and recovery after surgery. Ultimately, incorporating virtual reality into clinical practice may improve the hospital experience for children and their families and lead to better outcomes (25).

### 3.11. VR in breast cancer

A range of VR-based interventions improved various symptoms in breast cancer patients, such as anxiety, depression, pain, cognitive function, and upper limb movement, but not fatigue. With advancements in technological fields, VR methods are expected to become more commonly used and evolve. Several countries have already initiated adapting legislation and policy to support virtual reality technology and accelerate its integration into medical and healthcare practices (30).

### 3.12. AMSTAR-2 assessment

The AMSTAR-2 assessment is provided in Table 2. The overall confidence rated by the tool is moderate for two studies, low for two studies, and critically low for other studies. Most of the included reviews had described all the PICO components (domain 1). Most of the authors had registered the protocol before starting the review (domain 2). However, many reviews did not explain the selection of study designs (domain 3) or perform a comprehensive literature search (domain 4). Study selection (domain 5) and data extraction (domain 6) were performed in duplicate in almost half of the reviews. Most of the reviews did not provide a list of excluded studies (domain 7) or describe the included studies in adequate detail (domain 8). Risk of bias (RoB) assessment (domain 9) was partially addressed in most reviews while reporting on sources of funding across the included studies (domain 10) was often omitted. Data were synthesized (domain 11) using appropriate methods in most cases, but the potential impact of RoB in individual studies on the meta-analysis results (domains 12 and 13) was rarely assessed. A discussion of any heterogeneity observed in the results of the review (domain 14) was provided in a minority of the reviews. Similarly, only a few reviews investigated publication bias (domain 15). Lastly, most reviews declared a conflict of interest, including funding received for conducting the review (domain 16).

**Table 2:** A critical appraisal of included systematic reviews, (AMSTAR - “A MeaSurement Tool to Assess systematic Reviews”).


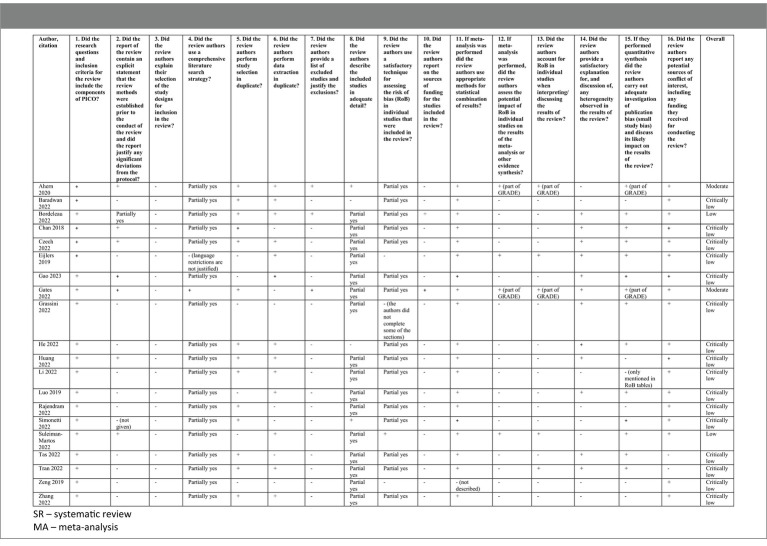

## 4. Discussion

The purpose of the current umbrella review was to offer a synthesis of SRs and MAs, which focused on the application of virtual reality in pain management.

All of the included SRs & MAs reported successful application of VR on pain relief in various situations including perioperative and periprocedural settings (orthopedic surgery and abdominal surgery) and for chronic pain (chronic low back pain, neck pain, fibromyalgia, phantom limb pain, hernia, rheumatology).

VR can be used as an alternative therapy for pain management in both children and adults, however, it appears to be more effective for children. VR therapy has been found to decrease pulse rate, anxiety, discomfort from pain, time spent thinking about the discomfort, and time taken to switch medication, but it has a negligible impact on enhancing pain tolerance. For acute pain, VR therapy can also effectively lower different types of pain, such as during postoperative dressing change, burn management, needle insertion, and thermal stimulation. However, VR therapy appears to be not sufficiently effective for chronic pain, which could be attributed to its limited ability to increase pain tolerance (22).

The suggested mechanisms of VR in pain management mainly include heightened distraction with gaining patient’s attention and activating many senses (3). Virtual environments use head tracking systems, tactile feedback and highly stimulating visual and auditory sensations. In this way, the individual becomes immersed in the virtual world, taking attention away from the perception of pain (3). It was hypothesized that the VR program, as a distractor, reduces the conscious sensation of pain through the signaling pathways leading to pain (9). Therefore, virtual reality may alter the behavior of the pain modulation system by lowering the concentration on pain resulting in a stimulus not being perceived as painful. By activating the visual cortex and integrating additional senses, virtual reality alters the handling of nociceptive stimuli. Brain functional magnetic resonance imaging (fMRI) confirms that virtual reality influences the insular and sensory cortex similar to opiates. Being expensive, VR systems have limited use, but with the increasing use of high-resolution mobile phones, VR has become a more adaptable and accessible tool for pain relief (10, 11).

Our findings are consistent with the results of other narrative reviews. Three reviews (31–33), similar to our results, suggested the effectiveness of VR in reducing anxiety, pain, and improving patient satisfaction in various procedures, surgeries, and critical setting, such as colonoscopies, trauma, burns, stroke, craniotomies, needle and dental procedures. VR demonstrated benefits for chronic pain as well (33). This evidence, although limited, indicates that VR might reduce the analgesia requirements across all age groups. Alanazi’s (2022) review (34) on pediatric oncology patients also corresponds with our findings about the successful application of VR in pain relief, as it was observed to serve as a distraction from painful procedures.

### 4.1. Implication for practice and research

#### 4.1.1. Back pain

Additional research is necessary to arrive at a conclusive statement about the effect of VR on chronic back pain. To assess the potential of VR-based therapies for long-term pain relief, it is crucial to conduct extended therapeutic and follow-up analyses. In the future, customized VR sessions could be provided to cater to the specific needs of different patients for optimal therapeutic effects. Additionally, further analyses should examine pain-related outcomes, including undesired effects, the return to work after a sick-leave, as well as mental health and overall well-being. Moreover, it is vital to determine the optimal exposure time for VR-based therapies. Future research could also focus on understanding the mechanisms that facilitate the use of VR-based therapies in chronic pain management. This could be accomplished by utilizing physiological and neuro-physiological indices (20). This will allow healthcare professionals to provide effective and evidence-based VR therapies to their patients. Furthermore, developing guidelines and best practices could aid in standardizing the application of VR to managing chronic pain, ensuring patient safety, and maximizing the effectiveness of the therapy. Overall, continued research into VR-based therapies for chronic pain management is necessary to fully understand their potential benefits and limitations. By investigating the mechanisms of action, examining different patient populations, and analyzing long-term outcomes, healthcare professionals can tailor VR therapy to fulfill the specific requirements of individual patients. In addition, developing guidelines and best practices could make VR-based therapies more accessible and effective for a broader range of patients (20).

#### 4.1.2. Pain during labor

Although, there is evidence that VR might be helpful for women during labor (11) to confirm these findings and provide stronger evidence, further randomized controlled trials with more participants and from multiple centers are necessary. Additionally, future studies should extend their follow-up period to examine the benefits of VR during labor. The effects of virtual reality on various aspects should be further investigated. These could include questions such as how satisfied the patients were, how long the first and second stages of labor lasted, and what quantities of intravenous analgesics they required. The frequency and duration of using VR to distract women during labor also need to be determined by upcoming trials. Furthermore, subsequent studies should examine the influence of laboring women’s characteristics and the visual element design on pain management. Additionally, the cost-effectiveness of VR in labor management should be analyzed. More research is necessary to identify whether a universally appropriate set of images for VR in pain management exists or if this should be a custom-made selection. Prior education and personalized VR content are encouraged to achieve fuller engagement from patients (10).

#### 4.1.3. VR for pain management during painful procedures

Although there is some early evidence supporting the effectiveness of VR for some specific painful procedures, the evidence quality is scarce and inconsistent. Therefore, future high-quality research is needed to validate the findings before VR can be widely adopted. Future studies should also investigate the cost-effectiveness of VR and explore its potential in other painful procedures (15).

#### 4.1.4. VR for phantom limb pain

VR was suggested to be effective in reducing phantom limb pain; however, due to the low number of studies with small sample sizes, more original studies are required. Furthermore, since some important aspects such as gender, the reason for amputation, the location of the amputation, or time spent since amputation have not been reported properly, future studies should address them.

VR is a new treatment method that enables patients to be fully immersed in a digital environment where their amputated limb is virtually created, providing the illusion that it is present. This is achieved by using a head-mounted display that places screens in front of the patient’s eyes, enhancing the sense of immersion.

#### 4.1.5. VR in pediatric patients

VR in pediatrics might be recommended to reduce their physical and psychological discomfort for painful medical interventions like a blood draw, minimally invasive dental procedures, or burn management (28). It is important for healthcare providers to choose distractors, such as VR, that are practical and appealing to the individual (17). However, due to insufficient evidence, it is unclear yet if VR-induced distraction can be used as a pain-reducing strategy for children with severe pain. Therefore, research should be focused on conducting large and well-designed trials that can provide meaningful results to guide clinical recommendations (19). It is worth mentioning that VR may not be suitable for certain pediatric patients, such as neonates or those with claustrophobia. Future studies should also focus on different age groups and surgical procedures, as well as the time and length of exposure to VR. Collaboration with VR designers to create age-appropriate applications may also be helpful. Additional studies could also investigate parents’ and guardians’ perspectives.

While VR has shown promise as an effective method for reducing anxiety in pediatric surgical patients, future studies are recommended to deeper examine its potential benefits and limitations. Collaboration with VR designers to devise age-appropriate applications and exploration of its potential for reducing postoperative pain are areas that require further investigation. Future studies are also required to optimize the use of such distraction-based interventions in preoperative and postoperative settings for children undergoing surgery (26).

#### 4.1.6. VR in cancer pain management

VR demonstrated reduced pain and anxiety in adult cancer survivors (29). However, due to the limitations of the original studies, specific recommendations and conclusions about breast cancer could not be drawn (30). Future large-scale pilot studies should be conducted to examine how feasible and applicable VR is for symptom management and rehabilitation in breast cancer patients.

#### 4.1.7. Strengths

We analyzed all available systematic reviews, which met the inclusion criteria and assessed their methodological quality using the AMSTAR guidelines. We tried to use the best practices for umbrella reviews. We interpreted the key findings and identified key areas of relevant to our diverse group of clinicians, researchers and stakeholders.

#### 4.1.8. Limitations

The main limitation of this review is that there are relatively few original studies, systematic reviews, and meta-analyses included in this umbrella review. Similarly, there is a low number of patients included in the original studies. Additionally, there is a high heterogeneity of pain syndromes varying from the acute procedure or surgery-related pain to chronic cancer-related pain included in the umbrella review, for this reason, we did not conduct a meta-analysis. Finally, most reviews had critically low confidence based on AMSTAR-2 assessment.

## 5. Conclusion

VR showed its efficacy for managing various pain conditions including acute pain during procedures and surgery-related pain as well as different chronic pain conditions, such as fibromyalgia, phantom limb syndrome, chronic low back and neck pain, and cancer-related pain conditions. VR might also be recommended to manage pain and anxiety in pediatric patients undergoing different painful procedures.

## Author contributions

DV: conceptualization, methodology, writing—original draft, and writing—review and editing. KT: data extraction, quality assessment, and writing—review and editing. MD, SS, and YGA: conceptualization and writing—review and editing. All authors contributed to the article and approved the submitted version.

## Funding

This work was supported in part by Nazarbayev University Faculty Development Competitive Research grant nos. SOM2021005 and 021220FD2851.

## Conflict of interest

The authors declare that the research was conducted in the absence of any commercial or financial relationships that could be construed as a potential conflict of interest.

## Publisher’s note

All claims expressed in this article are solely those of the authors and do not necessarily represent those of their affiliated organizations, or those of the publisher, the editors and the reviewers. Any product that may be evaluated in this article, or claim that may be made by its manufacturer, is not guaranteed or endorsed by the publisher.
